# Association of genes of protease-antiprotease balance pathway to lung function and emphysema subtypes

**DOI:** 10.1186/1471-2466-13-36

**Published:** 2013-06-04

**Authors:** Mari K Kukkonen, Emmi Tiili, Tapio Vehmas, Panu Oksa, Päivi Piirilä, Ari Hirvonen

**Affiliations:** 1 Finnish Institute of Occupational Health, Topeliuksenkatu 41 a A, FI-00250, Helsinki, Finland; 2 Department of Clinical Physiology, Helsinki University Hospital, Helsinki, Finland

**Keywords:** Emphysema, Lung function, Genetics, Protease-antiprotease balance

## Abstract

**Background:**

The imbalance between proteases and antiproteases has been proposed to participate to the pathogenesis of chronic obstructive pulmonary disease (COPD) and emphysema. Gene level variation in different metalloproteinases, metalloproteinase inhibitors, and cytokines affecting them may contribute to this imbalance and destruction of the lung parenchyma. We investigated whether polymorphisms in selected protease-antiprotease balance pathway genes predispose to different emphysema subtypes (centrilobular, paraseptal, panlobular, and bullae) and airflow limitation among Finnish construction workers.

**Methods:**

Eleven single nucleotide polymorphisms (SNPs) from seven genes (*GC*: rs7041 and rs4588; *MMP1*: rs1799750; *MMP9*: rs3918242; *MMP12*: rs652438; *TIMP2*: rs2277698; *TNF*: rs1799724 and rs1800629; *TGFB1*: rs1800469, rs1800470, and rs2241718) were analyzed from 951 clinically and radiologically characterized construction workers. The genotype and haplotype data was compared to different emphysematous signs confirmed with high resolution computed tomography (HRCT), forced vital capacity (FVC), forced expiratory volume in one second (FEV_1_), and maximal expiratory flow at 50% of FVC (MEF50) by using linear and logistic regression analyses, adjusted for potential confounders.

**Results:**

The *TIMP2* rs2277698 SNP was associated with overall (*p* = 0.022) and paraseptal (*p* = 0.010) emphysema, as well as with FEV_1_/FVC ratio (*p* = 0.035) and MEF50 (*p* = 0.008). The *TGFB1* rs2241718 and *MMP9* rs3918242 SNPs were associated with centrilobular emphysema (*p* = 0.022 and *p* = 0.008), and the *TNF* rs1800629 SNP with paraseptal emphysema (*p* = 0.017). In stratified analysis, individuals with at least one *TIMP2* rs227769*8* or *TNF* rs1800629 variant allele were found to be at around two-fold risk for pathological paraseptal changes (OR 1.94, 95% CI 1.14-3.30; OR 2.10, 95% CI 1.24-3.56). On the contrary, the risk for pathological centrilobular changes was halved for individuals with at least one *MMP9* rs3918242 (OR 0.51, 95% CI 0.30-0.86) or *TGFB1* rs2241718 (OR 0.53, 95% CI 0.30-0.90) variant allele, or *TGFB1* rs1800469-rs1800470 AT-haplotype (OR 0.55, 95% CI 0.33-0.93). MEF50, in turn, was significantly reduced among individuals with at least one *TIMP2* rs227769*8* variant allele (*p* = 0.011).

**Conclusion:**

Our findings strengthen the hypothesis of the importance of protease-antiprotease balance in pathogenesis of emphysema and shed light on the aetiology of different emphysema subtypes by associating *MMP9* and *TGFB1* to centrilobular emphysema, and *TIMP2* and *TNF* to paraseptal emphysema and/or airflow obstruction.

## Background

The imbalance between proteases and antiproteases is widely accepted as a mechanism behind the lung tissue destruction leading to pulmonary emphysema [1,2]. This theory has been building up since the discovery of severe deficiency of a serine protease inhibitor Alpha-1-antitrypsin (encoded by *SERPINA1* gene), which has been shown to predispose to early panlobular emphysema [3]. Subsequently, the gene of another member of the serpin-family, SERPINE2, has been associated to chronic obstructive pulmonary disease (COPD) and emphysema in several populations [4-7]. In agreement with this, our recent findings suggested that *SERPINE2* gene polymorphisms may be involved particularly in the development of panlobular emphysema [8].

In addition to the serine proteases, there are a number of proteolytic enzymes capable of degrading elastin and other matrix macromolecules, such as the matrix metaloproteinases (MMPs). MMPs play an essential role in tissue repair and remodeling, and there is increasing evidence that some of them may be important in airway inflammation and the development of COPD and emphysema [2]. It has been shown that transgenic mice over-expressing MMP1 or MMP9 develop pulmonary changes comparable to human emphysema [9,10], and that mice lacking the *MMP12* gene are protected from emphysema despite a long term exposure to cigarette smoke [11]. Furthermore, several polymorphisms in *MMP1*, *MMP9*, and *MMP12* genes have been associated to emphysema and related phenotypes [12-15].

Tissue inhibitors of metalloproteinases (TIMPs), in turn, may contribute to the disturbance of protease-antiprotease balance by binding MMPs and inhibiting their actions. In fact, variation in the *TIMP2* gene has been associated to the development of COPD in two different populations [16,17]. In addition, differences in the expression of TGFB1 and TNF cytokines have been shown to influence the pathogenesis of COPD in animal models, possibly via interactions with MMP9 and MMP12 [18-20]. Genetic association studies have connected several polymorphisms and/or haplotypes of *TGFB1* and *TNF* genes to the development of COPD [21-24], and recent meta-analyses have confirmed some of the associations [25,26].

GC (Group specific component; also known as Vitamin D-binding protein, VDBP) is a multifunctional protein, suggested to have a role in chronic inflammatory response in the lungs [27]. Certain polymorphisms in the *GC* gene have previously been associated to COPD [28,29].

We investigated whether polymorphisms in the *GC* gene (rs7041 and rs4588) and six genes of the protease-antiprotease balance (*MMP1*: rs1799750; *MMP9*: rs3918242; *MMP12*: rs652438; *TIMP2*: rs2277698; *TNF*: rs1799724 and rs1800629; *TGFB1*: rs1800469, rs1800470, and rs2241718) predispose to emphysematous changes and airflow limitation among Finnish Caucasian construction workers. These polymorphisms were selected based on previous association studies suggesting them as potential modifiers of the examined pulmonary disorders.

Signs of emphysema subtypes (centrilobular, paraseptal, panlobular, and bullae) were determined from all the study subjects by using the high-resolution computed tomography (HRCT) –technique. Lung function was measured by using flow volume spirometry.

## Methods

### Study population

This study combines data from two previous screening studies. The first study group (ASBE, n = 602) was recruited in 1996–1997 and consisted of asbestos exposed subjects who lived in Helsinki area, had asbestosis or asbestos-related pleural plaques, and had a positive smoking history [30,31]. The second study group (ASSE, n = 633) was recruited in 2003–2004 and consisted of asbestos exposed persons from three geographic areas (Helsinki, Tampere, Turku), who had previously been diagnosed with an asbestos related occupational disease, or had visited the clinics of occupational medicine in Helsinki, Turku or Tampere for a clinical follow-up [32].

Altogether 178 of the subjects recruited in 2003–2004 had already participated in the first study. They were therefore excluded from the second patient group in the present study before combining the data. In the combined study population, blood samples were available for 1021 subjects, 1013 of whom the genotype data were successfully achieved. However, 25 additional subjects were excluded because of missing smoking information and 37 because of insufficient asbestos exposure data. Thus, the final study group consisted of 951 subjects (935 males, 16 females).

An approval for the study was obtained from the local ethics committees (Ethical Committee of the Finnish Institute of Occupational Health and Ethics Committee for Research in Occupational Health and Safety, Hospital District of Helsinki and Uusimaa) according to the legislation at the time of the original study. All subjects gave an informed consent to participate in the study.

### Radiological examinations

The lungs of the construction workers were imaged prone in full inspiration with four different scanners: in 1996–1997 the Picker PO 2000 (Picker International, Cleveland, USA) device was used, whereas in 2003–2004 Siemens Somatom Balance (Siemens Medical, Erlangen, Germany) was used in Helsinki, Siemens Somatom Plus 4 (Siemens Medical) was used in Tampere, and GE Light-speed 16 Advantage (GE Healthcare, Milwaukee, WI, USA) was used in Turku.

The HRCT images were printed as hard copies and analyzed blindly by two (2003–2004) or three (1996–1997) radiologists. Emphysema was defined as sharply delineated low-density area(s) according to the criteria and reference images given by Webb *et al*. [33]. The radiologic signs of centrilobular, paraseptal, panlobular, and bullae-type emphysema were scored in both lungs by using a scale from 0 to 5 (the emphysema subtype score): 0 (no changes), 1 (faint or subnormal abnormalities, in a single slice or few slices), 2 (slight abnormalities in some slices), 3 (clear abnormalities in several slices), 4 (score between 3 and 5), and 5 (abnormalities widely distributed in the whole lung, in all or most slices). These emphysema subtype scores were added up to form the emphysema sum score, its maximum being 20 per lung [31]. Mean scores of both lungs were used in the analysis. The intra- and inter-reader consistencies of readings have previously been reported [30].

### Lung function examinations

Flow-volume spirometry was performed with a rolling-seal spirometer (Mijnhard BV, Bunnik, Holland) connected to microcomputer (Medikro MR-3; Medikro, Kuopio, Finland), using Finnish reference values [34] and the standards of the European Respiratory Society (ERS) [35]. The following parameters were measured: forced vital capacity (FVC), forced expiratory volume in 1 second (FEV_1_), the FEV_1_/FVC ratio (FEV_1_/FVC × 100), and the maximal expiratory flow where 50% of FVC remains exhaled (MEF50).

The FVC, FEV_1_, and MEF50 were handled as percent of Finnish reference values [34] based on the distribution of values in the reference population. The FEV_1_ and FVC values were considered decreased if they were <80% of predicted, FEV_1_/FVC ratio if it was <88% of predicted, and MEF50 if it was <62% of predicted [34].

### Genotyping analyses

DNA was extracted mechanically (Thermo King Fisher; Thermo Fisher Scientific, Erembodegem, Belgium) from whole blood using Biosprint 15 DNA Blood Kit (Qiagen GmbH, Hilden, Germany) and stored at −20°C until use.

Two *TNF* SNPs (rs1799724 and rs1800629), two *TGFB1* SNPs (rs1800469 and rs2241718), two *GC* SNPs (rs7041 and rs4588), one *MMP12* SNP (rs652438), and one *TIMP2* SNP (rs2277698) were genotyped by using the OpenArray-system (BioTrove Inc., Woburn, MA, USA), a next-generation quantitative PCR platform based on TaqMan chemistry. The assay ID:s for TaqMan® SNP Genotyping Assays spotted on the array were C__11918223_10, C___7514879_10, C___8708473_10, C___7818377_1_, C___3133594_30, C___8278879_10, C____785907_10, and C__15885241_10, respectively. Plate format of 16 SNPs and 144 samples per array was used. The allele calling analysis was performed by using OpenArray™ SNP Genotyping Analysis software (BioTrove Inc.).

The third analysed *TGFB1* SNP (rs1800470) was genotyped by using an allelic discrimination assay on the ABI 7500 Real-Time PCR system (Applied Biosystems, Foster City, CA, USA) with TaqMan® probes [36]. The primers and probes used in the assay were as follows: forward primer: 5′-GCG CTC TCG GCA GTG C-3′; reverse primer: 5′-CCA GGC GTC AGC ACC AGT A-3′; VIC-probe: 5′-AGC AGC GGC AGC A-3′; and FAM-probe: 5′-CAG CAG CAG CAG C-3′. The primer and probe concentrations in the PCR reaction were 1200 nM and 200 nM, respectively, and the cycling conditions were 50°C for 2 minutes, 95°C for 10 minutes, 40 cycles of 95°C for 15 seconds, and 62°C for 1 minute. Sequence Detection Software 1.4 (Applied Biosystems) was used for the allele calling analysis.

The *MMP1* SNP (rs1799750) was analysed with a pyrosequencing-method based on an assay from PyroMark Assay Database (Qiagen). The PCR-primers were: forward primer 5′-biotin-CCC TTA TGG ATT CCT GTT TTC-3′; reverse primer: 5′-CCC ATT CTT CTT ACC CTC TTG-3′.

The primer concentrations in PCR reactions were 500 nM, and the cycling conditions were: 95°C for 5 minutes, 35 cycles of 95°C for 30 seconds, 54°C for 30 seconds, and 72°C for 30 seconds followed by a final extension of 72°C for 5 minutes. The pyrosequencing run was performed with PSQ™96MA (Qiagen) by using Pyromark Gold Q96 Reagents (Qiagen) according to manufacturer’s recommendations. Briefly, 40 μl of the PCR product was mixed with 37 μl of Binding buffer and 3 μl of Streptavidin Sepharose High Performance beads (GE Healthcare, Uppsala, Sweden). PCR products bound to the beads were collected and denatured to single-stranded by treatment with 70% Ethanol (Aa), Denaturation Buffer, Washing Buffer, and mQ water in Pyrosequencing Washing Station. The sequencing primer 5′-GTA GTT AAA TAA TTA GAA AG-3′ was attatched to the template by incubating for 2 minutes in 80°C in annealing buffer. The Pyrosequencing run was conducted in the dispensation order of CAGCTACTAGCA. The pyrograms were generated and analyzed with PSQ 96 SNP Software 1.1.

The *MMP9* SNP (rs3918242) was genotyped by using a PCR-RFLP-based method essentially according to Joos *et al*. [37]. Briefly, the concentrations of the forward (5′-TTC GTG ACG CAA AGC AGA-3′) and reverse (5′-AGC AGC CTC CCT CAC TCC T-3′) primers were 670 nM, and the cycling conditions were 95°C for 4 minutes, 34 cycles of 95°C for 30 seconds, 58°C for 30 seconds, and 72°C for 45 seconds followed by a final extension of 72°C for 5 minutes. After digestion with *Sph*I in 37°C for 3 hours the PCR product was electrophoresed on a 2% agarose gel containing EtBr and visualized under UV-light.

For the quality control, two independent readers interpreted the results and a random selection of 10% of all samples was re-tested. No discrepancies were discovered in the replicate tests for rs7041, rs4588, rs652438, rs1800470, rs1799750, and rs3918242. Minor error rates were detected for OpenArray assays rs1799724 (1%), rs1800629 (2%), rs2241718 (2%), rs2277698 (2%), and rs1800469 (3%).

To verify the reliability of OpenArray platform, a random selection of 15% of samples was re-analyzed for rs1800629 and rs1799724 with 100% concordant results with the earlier estimates.

The re-analyses were conducted with an RFLP-based method [38] (rs1800629) and a pyrosequencing-based method (rs1799724). The primers and probes for the pyrosequencing protocol designed by using PyroMark Assay Design 1.0 -tool (Qiagen) were as follows: forward primer: 5′-GGT AGG AGA ATG TCC AGG GCT ATG-3′, biotinylated reverse primer: 5′-biotin-ACT CCC TGG GGC CCT CTA-3′ and sequencing primer: 5′-TCG AGT ATG GGG ACC-3′. The primer concentrations in PCR reactions were 200 nM, and the cycling conditions were: 95°C for 5 minutes, 39 cycles of 95°C for 15 seconds, 56°C for 30 seconds, and 72°C for 15 seconds followed by a final extension of 72°C for 5 minutes. The pyrosequencing was performed with PSQ™96MA (Qiagen) by using Pyromark Gold Q96 Reagents (Qiagen) as described above for the analysis of the *MMP1* rs1799750 SNP.

### Statistical analysis

Our study (n = 951) has 80% power to detect odds ratios (ORs) from 1.46 to 2.30 depending on the minor allele frequency (4-42%). The calculations, based on a two-sided alpha of 0.05, were performed by using standard methods.

The χ^2^ analysis with a cut-off p-value of 0.05 was used to test for a deviation from the Hardy-Weinberg equilibrium (HWE).

The linkage disequilibrium (LD) structure was examined by using HaploView program, version 4.2 [39]. When moderate or strong linkage was detected (r^2^ > 0.5), haplotypes consisting of the SNPs in question were statistically reconstructed from population genotype data with the Markov chain method for haplotype assignments by using the PHASE program (version 2.1) [40]. The associations of the haplotypes to pulmonary parameters were then examined as with the single SNPs.

The associations between genotypes/haplotypes, emphysema, and lung function parameters (FEV_1_, FVC, FEV_1_/FVC and MEF50) were evaluated by using general linear model, whereas logistic regression analysis was used to evaluate the potential confounders and to further study the risk for emphysematous changes and their severity with certain genotype. For further analysis, the cases were divided according to the existence of radiologic changes (Table 1). The radiologic signs of emphysema were then considered either subnormal if the emphysema subtype score was <1 (<2 in emphysema sum score), or pathological if the emphysema subtype score was ≥1 (≥2 in emphysema sum score).

**Table 1:** Distribution of radiologic signs in relation to emphysema subtypes

	**Radiologic signs**			
	**No changes**	**Any changes**	Subnormal changes^*^	**Pathological changes**^**†**^
	**n (%)**	**n (%)**	**n (%)**	**n (%)**
Emphysema^‡^	599 (63.0)	352 (37.0)	231 (24.3)	121 (12.7)
Centrilobular	723 (76.0)	228 (24.0)	105 (11.0)	123 (13.0)
Paraseptal	781 (82.1)	170 (17.9)	94 (9.9)	76 (8.0)
Panlobular	783 (82.3)	168 (17.7)	109 (11.5)	59 (6.2)
Bullae	826 (86.9)	125 (13.1)	90 (9.4)	35 (3.7)

^*^The changes were considered as subnormal if the radiologic score was < 1 (< 2 in emphysema sum score).

^†^The changes were considered as pathological if the radiologic score ≥ 1 (≥ 2 in emphysema sum score).

^‡^The sum score of all emphysematous changes.

Covariates used in the analysis were: sex, age, pack years of smoking (PYs) and years of asbestos exposure for emphysema, and PYs and years of asbestos exposure for FEV_1_, FVC, FEV_1_/FVC ratio, and MEF50.

All of the data analyses were performed by using the SPSS version 18.0 (SPSS Inc., Chicago, IL).

## Results

The demographics, pulmonary function data, and HRCT characteristics of the construction workers are summarized in Table 2. The genotype frequencies of the studied SNPs among subjects with different type of emphysematous changes are shown in an additional table [see Additional file 1: Table S1]. All the genotype distributions of the studied gene polymorphisms were in HWE in the whole study population (*p* > 0.09).

**Table 2:** Selected characteristics of the construction workers

	**Mean (SD) or N (%)**
**Age, years**	63.2 (7.3)
**Male sex**	935 (98.3%)
**Smoking history**	
Never smoker	135 (14.2%)
Ex-smoker	595 (62.6%)
Current smoker	221 (23.2%)
Pack years	20.4 (16.7)
**Years of asbestos exposure (n = 943)**	23.9 (10.7)
Emphysema score (n = 352^*^)	2.00 (2.38)
Centrilobular (n = 228)	1.23 (1.01)
Paraseptal (n = 170)	1.00 (0.91)
Panlobular (n = 168)	0.93 (0.87)
Bullae (n = 126)	0.70 (0.77)
**Lung function**	
FEV_1_ (n = 925)	83.4 (18.6)
FVC (n = 921)	89.1 (15.9)
FEV_1_/FVC ratio (n = 921)	93.5 (12.8)
MEF50 (n = 923)	65.9 (29.5)

FEV_1_ = Forced expiratory volume in 1 second, % predicted; FVC = forced vital capacity, % predicted; FEV_1_/FVC ratio = FEV_1_/FVC x 100; MEF50 = maximal expiratory flow at 50% of forced vital capacity, % predicted; SD = standard deviation, n = 951 except as otherwise noted.

^*^Subjects with emphysema sum score >0.

The *TIMP2* rs2277698 was associated with emphysema sum score (*p* = 0.022) and paraseptal emphysema (*p* = 0.010). The *TGFB1* rs2241718 and *MMP9* rs3918242 SNPs were associated with centrilobular emphysema (*p* = 0.022 and *p* = 0.008, respectively), and the *TNF* rs1800629 SNP was associated with paraseptal emphysema (*p* = 0.017) (Table 3).

**Table 3:** **Association between genetic polymorphisms**, **emphysema and pulmonary function**

**Phenotype**	**Gene**	**Polymorphism**	β^*^	***p***-**value**
**Emphysema**				
All	*TIMP2*	rs2277698 (G/A)	0.071	0.022
Centrilobular	*TGFB1*	rs2241718 (G/A)	−0.071	0.022
	*MMP9*	rs3918242 (C/T)	−0.082	0.008
Paraseptal	*TNF*	rs1800629 (G/A)	0.075	0.017
	*TIMP2*	rs2277698 (G/A)	0.081	0.010
**FEV**_**1**_**/FVC ratio**	*TIMP2*	rs2277698 (G/A)	−0.066	0.035
**MEF50**	*TIMP2*	rs2277698 (G/A)	−0.084	0.008

FEV_1_/FVC ratio = FEV_1_/FVC x 100; MEF50 = maximal expiratory flow at 50% of forced vital capacity, % predicted; Covariates used in the analysis: sex, age, PYs, and years of asbestos exposure for emphysema scores; pack-years and years of asbestos exposure for MEF50.

^*^Standardized coefficient β.

The above mentioned association between *TIMP2* rs2277698 SNP and emphysema sum score did not come up in stratified analysis. Instead, the carriage of at least one *TIMP2* rs2277698 variant A-allele was found to pose a twofold risk for pathological paraseptal emphysema (OR 1.94, 95% CI 1.14-3.30) (Table 4). In addition, the carriage of at least one *TNF* rs1800629 variant A-allele was found to pose a twofold risk for overall (OR 2.03, 95% CI 1.38-2.98), subnormal (OR 2.12, 95% CI 1.30-3.48), and pathological (OR 2.10, 95% CI 1.24-3.56) paraseptal changes. In contrast, the carriage of at least one *TGFB1* rs2241712 variant A-allele was found to halve the risk for centrilobular emphysema (OR 0.53, 95% CI 0.30-0.90), as was also the carriage of at least one *MMP9* rs3918242 variant T-allele (OR, 0.51 95% CI 0.30-0.86) (Table 4).

**Table 4:** **Distribution of *TIMP2***, ***TGFB1***, ***MMP9***, **and *TNF *genotypes according to the existence and severity of emphysema changes**

**Emphysema phenotype**	**Genotype**	No radiologic changes^*^	**Radiologic changes**	**OR (95% CI)**^**†**^	**Subnormal^‡^ changes**	**OR (95% CI)**^**†**^	**Pathological^§^ changes**	**OR (95% CI)**^**†**^
		**n (%)**	**n (%)**		**n (%)**		**n (%)**	
**All**	*TIMP2* rs2277698							
	G/G	462 (77.9)	270 (77.8)	1.0	180 (79.6)	1.0	90 (74.4)	1.0
	G/A or A/A	131 (22.1)	77 (22.2)	1.02 (0.72-1.44)	46 (20.4)	0.92 (0.62-1.38)	31 (25.6)	1.35 (0.83-2.22)
**Centrilobular**	*TGFB1* rs2241718							
	G/G	518 (72.8)	173 (77.6)	1.0	74 (71.2)	1.0	99 (83.2)	1.0
	G/A or A/A	194 (27.2)	50 (22.4)	0.76 (0.52-1.11)	30 (28.8)	1.08 (0.68-1.73)	20 (16.8)	0.53 (0.30-0.90)
	*MMP9* rs3918242							
	C/C	519 (72.5)	171 (75.7)	1.0	74 (70.5)	1.0	97 (80.2)	1.0
	C/T or T/T	197 (27.5)	55 (24.3)	0.77 (0.53-1.12)	31 (29.5)	1.08 (0.68-1.72)	24 (19.8)	0.51 (0.30-0.86)
**Paraseptal**	*TIMP2* rs2277698							
	G/G	609 (78.9)	123 (73.2)	1.0	72 (78.3)	1.0	51 (67.1)	1.0
	G/A or A/A	163 (21.1)	45 (26.8)	1.41 (0.94-2.13)	20 (21.7)	1.04 (0.59-1.81)	25 (32.9)	1.94 (1.14-3.30)
	*TNF* rs1800629							
	G/G	605 (78.3)	111 (66.1)	1.0	61 (66.3)	1.0	50 (65.8)	1.0
	G/A or A/A	168 (21.7)	57 (33.9)	2.03 (1.38-2.98)	31 (33.7)	2.12 (1.30-3.48)	26 (34.2)	2.10 (1.24-3.56)

Data are presented as n (%) except as noted.

^*^Reference category.

^†^Adjusted for age, sex, pack-years and years of asbestos exposure.

^‡^The changes were considered as subnormal if the emphysema subtype score was < 1 (< 2 in emphysema sum score).

^§^The changes were considered as pathological if the emphysema subtype score was ≥ 1 (≥ 2 in emphysema sum score).

The *TIMP2* rs2277698 was also found to be associated with the FEV_1_/FVC ratio (*p* = 0.035) and MEF50 (*p* = 0.008). In further analysis, subjects with at least one *TIMP2* rs2277698 variant A-allele were found to have significantly lower MEF50 than subjects with homozygous *TIMP2* rs2277698 wild type genotype (61.0 ± 27.0 vs. 67.0 ± 29.9, *p* = 0.011). Similarly, the FEV_1_/FVC ratio tended to be lower among subjects homozygous to the *TIMP2* rs2277698 variant A-allele compared to the wild type genotype (89.1 ±9.3 vs. 93.8 ± 12.7, *p* = 0.136).

When gene-gene interactions were examined, a combination of homozygous variant allele genotypes of *TGFB1* rs2241718 and *MMP9* rs3918242 loci was found to reduce the risk of pathological centrilobular changes into fifth compared to the wild type genotypes (OR 0.22, CI 95% 0.08-0.61) (Table 5).

**Table 5:** **Distribution of*MMP9***/***TGFB1*combined genotypes according to the existence and severity of centrilobular changes**

**Genotype combination (*MMP9 *rs3918242-*TGFB1 *rs2241718)**	No radiologic changes^*^	**Radiologic changes**	**OR (95% CI)**^**†**^	**Subnormal^‡^ changes**	**OR (95% CI)**^**†**^	**Pathological^§^ changes**	**OR (95% CI)**^**†**^
	**n (%)**	**n (%)**		**n (%)**		n (%)^*^	
**Wild type-wild type**^**#**^	375 (52.8)	130 (58.3)	1.0	52 (50.0)	1.0	78 (65.5)	1.0
**Heterozygote/ homozygote**^**+**^	281 (39.6)	81 (36.3)	0.83 (0.60-1.15)	43 (41.3)	1.07 (0.69-1.68)	38 (31.9)	0.66 (0.43-1.02)
**Heterozygotes or homozygotes**^**±**^	54 (7.6)	12 (5.4)	0.63 (0.36-1.09)	9 (8.7)	1.29 (0.68-2.46)	3 (2.5)	0.22 (0.08-0.61)

^*^Reference category.

^†^Adjusted for age, sex, pack-years and years of asbestos exposure.

^‡^The changes were considered as subnormal if the emphysema subtype score was < 1 (< 2 in emphysema sum score).

^§^The changes were considered as pathological if the emphysema subtype score was ≥ 1 (≥ 2 in emphysema sum score).

^#^*MMP9 CC*/*TGFB1 GG.*

^+^*MMP9 CT*/*TGFB1* GG or *MMP9 CC*/*TGFB1 GA.*

^±^*MMP9 CT*/*TGFB1 GA* or *MMP9 TT* or *TGFB1 AA.*

In the linkage analyses, linkage disequilibrium was observed between the *GC* rs4588 and rs7041 SNPs (D’ = 1.00, r^2^ = 0.501). The *TGFB1* rs1800469 and rs1800470 SNPs were also linked to each other (D’ = 0.970, r^2^ = 0.738), but not with the third studied *TGFB1* SNP (rs2241712) (D’ <0.02 and r^2^ = 0.0). The *TNF* rs1799724 minor allele frequency was too small 0.2%) for r^2^ to detect LD, despite of the maximum D’ (D’ = 1.00, r^2^ = 0.008).

Haplotype analysis identified three haplotypes for *GC* rs4588 and rs7041 SNPs; the most common haplotype (65.2%) was GC (wild type-wild type), followed by GA (13.7%), and TA (21.1%) haplotypes. No associations were seen between the *GC* haplotypes and the studied parameters.

For *TGFB1* rs1800469 and rs1800470 SNPs four haplotypes were identified: GT (wild type-wild type, 60.5%), GC (24.9%), AT (10.3%), and AC (4.3%). The *TGFB1* haplotype (rs1800469-rs1800470) was found to be associated with centrilobular emphysema (*p* = 0.018) (data not shown). Moreover, in the stratified analysis the AT haplotype was found to almost halve the risk for centrilobular emphysema (OR 0.55, 95% CI 0.33-0.93) compared to the most common haplotype (GT) (Table 6).

**Table 6:** **Distribution of*TGFB1*haplotypes according to the existence and severity of centrilobular changes**

Haplotype^*^	**No radiologic changes**	**Radiologic changes**	**OR (95% CI)**^**‡**^	**Subnormal^§^ changes**	**OR (95% CI)**^**‡**^	**Pathological^#^ changes**	**OR (95% CI)**^**‡**^
	**n (%)**^**†**^	**n (%)**^**†**^		**n (%)**^**†**^		**n (%)**^**†**^	
**GT**	834 (57.7)	283 (62.1)	1.0	125 (59.5)	1.0	158 (64.2)	1.0
**GC**	398 (27.5)	119 (26.1)	0.88 (0.68-1.14)	52 (24.8)	0.91 (0.64-1.30)	67 (27.2)	0.90 (0.65-1.26)
**AT**	187 (12.9)	50 (11.0)	0.79 (0.55-1.13)	30 (14.3)	1.10 (0.71-1.72)	20 (8.1)	0.55 (0.33-0.93)
**AC**	27 (1.9)	4 (0.9)	0.44 (0.15-1.32)	3 (1.4)	0.72 (0.21-2.44)	1 (0.4)	0.22 (0.03-1.70)

^*^Composed of rs1800469 and rs1800470 SNPs.

^†^Data are presented as number of chromosomes (%).

^‡^Adjusted for age, sex, pack-years and years of asbestos exposure.

^§^The changes were considered as subnormal if the emphysema subtype score was <1 (< 2 in emphysema sum score).

^#^The changes were considered as pathological if the emphysema subtype score was ≥1 (≥ 2 in emphysema sum score).

## Discussion

We found a significant association between *MMP*9 rs3918242 variant T-allele and a lowered proneness to centrilobular emphysema. This contrasts the previous findings suggesting the T-allele as a risk factor for COPD and emphysema that is dominant in the upper lung [12,13]. However, similarly to our study, a recent Korean study with a reasonable study size found the T-allele protective against COPD [41].

In addition to *MMP9*, several animal and genetic studies have connected *MMP1* and *MMP12* to COPD and emphysema [9,11,42]. We did not, however, find any associations between the *MMP1* and *MMP12* SNPs and emphysema or lung functions decline.

The previous studies on *TGFB1* polymorphisms, COPD, emphysema, and related traits have provided contradictory results; some studies have found the variant alleles to predispose to emphysema and severe airflow limitation [23,24] while others have found them to protect against COPD [21,25].

In the current study, the *TGFB1* rs2241718 SNP and a haplotype consisting of the rs1800469 and rs1800470 SNPs were found to be associated with centrilobular emphysema. Stratified analysis showed that the variant A-allele in rs2241718 locus and a haplotype consisting of rs1800469 variant A-allele and rs1800470 wild-type T-allele were protective against pathological centrilobular changes. Together with the *MMP*9 rs3918242 variant T-allele the *TGFB1* rs2241718 variant A-allele reduced the risk of pathological centrilobular emphysema into fifth compared to the wild-type genotype.

Both TGFB1 and MMP9 can be secreted from alveolar macrophages, which are activated by cigarette smoke and found in increased number in airways and lung parenchyma of COPD patients [1]. Centrilobular emphysema, on the other hand, is the most common type of pulmonary emphysema and closely associated to cigarette smoke [43,44]. Hence, the effects of *TGFB1* and *MMP9* polymorphisms in the development of centrilobular disease could be mediated through macrophages via interaction with cigarette smoke. The studied SNPs in *TGFB1* and *MMP9* are also in strong linkage (r^2^ > 0.8 in 1000 Genomes Project population) with several other polymorphisms, and it is therefore possible that the causal variant resides in a completely different gene.

Although *TIMP2* polymorphisms have previously been connected to COPD [16,17], their association to different emphysema subtypes has remained unexplored. In our study, the *TIMP2* rs2277698 SNP was associated with overall and paraceptal emphysema, FEV_1_/FVC ratio, and MEF50. Stratified analysis revealed a twofold risk for pathological paraseptal changes for individuals with at least one variant A-allele. In addition, FEV_1_/FVC ratio tended to be lower among individuals homozygous with variant A-allele, and MEF50 was significantly reduced among individuals with at least one variant A-allele. Decreased FEV_1_/FVC ratio and MEF50 suggests obstruction in peripheral airways typical for COPD and smoking related emphysema [45].

The rs2277698 SNP is a synonymous base-substitution with unknown functional consequences. Although it has previously been speculated to associate with down regulation of TIMP2 activity leading to matrix degradation and COPD [16], this has remained unconfirmed.

The F-SNP program, connected to main databases (http://compbio.cs.queensu.ca/F-SNP/) [46], predicts that rs2277698 SNP is highly likely involved in splicing regulation. The rs2277698 is also in strong linkage (r^2^ > 0.9 in 1000 Genomes Project population) with other SNPs, some of which (rs9889410 and rs11654470) reside in an area predicted to alter the transcriptional regulation [46].

We also found an association between the *TNF* rs1800629 SNP and paraseptal emphysema. Further analysis revealed a twofold risk for pathological paraseptal changes for individuals with at least one variant A-allele. This finding is in agreement with a recent meta-analysis with over 5500 COPD patients and controls [25], while another meta-analysis suggests that the risk of developing COPD is statistically significant only among Asian subjects [26].

Since the rs1800629 variant A-allele has been shown to enhance the expression of TNF [47], and since the over-expression of TNF has been shown to induce emphysematous changes in mouse models [20,48], our findings support the role of *TNF* polymorphisms in the development of pulmonary emphysema, and their involvement in the pathogenesis of the paraseptal disease.

Certain genotypes and haplotypes of the multifunctional GC-protein, suggested to have a role in macrophage activation and chronic inflammatory response in the lungs, has been associated to COPD in several studies [28,29]. We did not, however, find any associations between these particular genotypes or haplotypes and emphysema subtypes or lung function.

One of the main strengths of our study is that lung function and CT-defined emphysema subtypes were recorded separately and classified according to their severity; it is highly likely that the disease pathogenesis vary between different subgroups. Another advantage is that our patient material was considerably large and a lot of ex- and current smokers were included. This is useful in demonstrating the genetic predisposition to emphysema, which probably would not have manifested to such degree without smoking.

Our study also has some potential limitations. First, since the patients were enrolled in three cities during two separate primary studies, four different CT-scanners were used and seven radiologists participated in the image reading. However, since the Finnish population is very homogenous and the three cities, where the patients were enrolled, are all located in the southern Finland very close to each other, we do not believe that the geographic origin has caused any significant bias in the data analysis. Moreover, any inconsistency in image reading or in the technical image quality causes inaccuracy and thus random noise to the results leading in loss of power rather than in a systematic error. This increases the error variance in computations and the detected associations are therefore likely to be underestimated.

Second, our study subjects have been selected based on their asbestos exposure, which itself appeared not to be a significant predictor for emphysematous changes in the logistic regression model (data not shown). However, it is highly likely that the study subjects have also been occupationally exposed to other particles, such as concrete, silica, and wood dusts, which can contribute to the development of emphysema. Unfortunately the exposure data of other dusts was not available from our study subjects. However, like tobacco smoke, exposure to these substances is likely to promote the detection of genetic predisposition to emphysema.

Third, the multiple comparisons performed increase the possibility of detecting false-positive associations. However, most of the methods correcting for multiple testing are very conservative, and it is not clear, *e*.*g*., what is the correct number of comparisons one should adjust for [49]. In addition, based on previous findings, we had an *a priori* hypothesis for each polymorphism chosen, which reduces the need for such correction. Yet, these results should be considered with caution until replicated in another study population.

## Conclusions

To conclude, our findings support the hypothesis of the importance of protease-antiprotease balance in pathogenesis of emphysema and shed light on the aetiology of different emphysema subtypes. In particular, polymorphisms in *MMP9* and *TGFB1* are proposed to protect against centrilobular emphysema, and polymorphisms in *TIMP2* and *TNF* seem to increase the risk for paraseptal emphysema and/or airflow obstruction.

## Abbreviations

AAT: Alpha-1-antitrypsin; ASBE: Patient group recruited in 1996–1997; ASSE: Patient group recruited in 2003–2004; COPD: Chronic obstructive pulmonary disease; CT: Computed tomography; GC: Group specific component; FEV1: Forced expiratory volume in 1 second, % predicted; FVC: Forced vital capacity, % predicted; HRCT: High resolution computed tomography; HWE: Hardy-Weinberg equilibrium; LD: Linkage disequilibrium; MEF50: Maximal expiratory flow where 50% of FVC remains exhaled; MMP: Matrix metalloproteinase; MMP1: Matrix metalloproteinase 1 (interstitial collagenase); MMP9: Matrix metalloproteinase 9 (gelatinase); MMP12: Matrix metalloproteinase 12 (macrophage elastase); PY: Pack-years; SERPINA1: Serpin peptidase inhibitor, clade A, member 1; SERPINE2: Serpin peptidase inhibitor, clade E, member 2; SNP: Single nucleotide polymorphism; TGFB1: Transforming growth factor, beta 1; TIMP2: Tissue inhibitor of metalloproteinases 2; TNF: Tumor necrosis factor; VDBP: Vitamin D binding protein (see also GC).

## Competing interests

The authors declare that they have no competing interests.

## Authors' contributions

MKK carried the main responsibility of the genotyping, data analyses, and preparation of the manuscript; ET participated in the genotyping analyses and manuscript preparation; TV participated in the data collection, radiological examinations, data analysis, and manuscript preparation; PO participated in the data collection and manuscript preparation; PP participated in data collection, planning and analysis of lung function examinations, and manuscript preparation; AH was responsible for the study design and supervision of the genotyping, data analysis, and the manuscript preparation. All authors have read and approved the final version of the manuscript.

## Pre-publication history

The pre-publication history for this paper can be accessed here:

http://www.biomedcentral.com/1471-2466/13/36/prepub

## Supplementary Material

Additional file 1: Table S1 Genotype frequencies of the studied SNPs. Click here for file
