# Supplementary material for: Association of genes of protease-antiprotease balance pathway to lung function and emphysema subtypes
Source: BMC Pulm Med. 2013 Jun 4;13:36. doi: 10.1186/1471-2466-13-36 (PMC3680142; doi:10.1186/1471-2466-13-36)
Supplement: Additional file 1: Table S1 — Genotype frequencies of the studied SNPs. [file 1471-2466-13-36-S1.pdf]

Additional table 1 Genotype frequencies of the studied SNPs among Finnish construction workers according to the existence of different emphysematous changes

				Radiologic signs of emphysema					
**Gene**	**SNP**	**Genotypes**	**Amino acid change**	**No changes n (%)**	**Any changes n (%)**	**Centrilobular n (%)**	**Paraceptal n (%)**	**Panlobular n (%)**	**Bullae n (%)**
GC	rs7041	TT	Glu432Glu	254 (42.8)	136 (39.2)	83 (36.7)	63 (37.5)	59 (35.3)	54 (43.9)
	(n=941)[a]	TG	Glu432Asp	278 (46.8)	161 (46.4)	113 (50.0)	80 (47.6)	82 (49.1)	57 (46.3)
		GG	Asp432Asp	62 (10.4)	50 (14.4)	30 (13.3)	25 (14.9)	26 (15.6)	12 (9.8)
	rs4588	CC	Thr436Thr	365 (61.4)	216 (62.4)	133 (59.1)	98 (58.7)	105 (62.9)	79 (64.2)
	(n=940)[a]	CA	Thr436Lys	204 (34.3)	117 (33.8)	86 (38.2)	64 (38.3)	56 (33.5)	43 (35.0)
		AA	Lys436Lys	25 (4.2)	13 (3.8)	6 (2.7)	5 (3.0)	6 (3.6)	1 (0.8)
MMP1	rs1799750	GG		209 (35.1)	109 (31.3)	67 (29.6)	42 (24.9)	54 (32.3)	35 (28.5)
	(n=944)[a]	G-		272 (45.6)	171 (49.1)	108 (47.8)	90 (53.3)	84 (50.3)	65 (52.8)
		--		115 (19.3)	68 (19.5)	51 (22.6)	37 (21.9)	29 (17.4)	23 (18.7)
MMP9	rs3918242	CC		428 (72.1)	262 (75.3)	171 (75.7)	124 (73.4)	123 (73.7)	91 (74.0)
	(n=942)[a]	CT		154 (25.9)	82 (23.6)	52 (23.0)	44 (26.0)	43 (25.7)	31 (25.2)
		TT		12 (2.0)	4 (1.1)	3 (1.3)	1 (0.6)	1 (0.6)	1 (0.8)
MMP12	rs652438	AA	Asn357Asn	549 (92.4)	324 (93.4)	210 (92.9)	155 (92.3)	159 (95.2)	116 (94.3)
	(n=941)[a]	AG	Asn357Ser	45 (7.6)	23 (6.6)	16 (7.1)	13 (7.7)	8 (4.8)	7 (5.7)
		GG	Ser357Ser	0 (0.0)	0 (0.0)	0 (0.0)	0 (0.0)	0 (0.0)	0 (0.0)
TIMP2	rs2277698	GG		462 (77.9)	270 (77.8)	175 (77.4)	123 (73.2)	125 (74.9)	93 (75.6)
	(n=940)[a]	GA		123 (20.7)	68 (19.6)	45 (19.9)	39 (23.2)	37 (22.2)	27 (22.0)
		AA		8 (1.3)	9 (2.6)	6 (2.7)	6 (3.6)	5 (3.0)	3 (2.4)
TNF	rs1799724	CC		530 (89.2)	308 (89.3)	200 (89.3)	152 (90.5)	149 (89.2)	114 (92.7)
	(n=939)[a]	CT		62 (10.4)	37 (10.7)	24 (10.7)	16 (9.5)	18 (10.8)	9 (7.3)
		TT		2 (0.3)	0 (0.0)	0 (0.0)	0 (0.0)	0 (0.0)	0 (0.0)
	rs1800629	GG		465 (78.3)	251 (72.3)	163 (72.1)	111 (66.1)	119 (71.3)	90 (73.2)
	(n=941)[a]	GA		120 (20.2)	87 (25.1)	60 (26.5)	52 (31.0)	42 (25.1)	31 (25.2)
		AA		9 (1.5)	9 (2.6)	3 (1.3)	5 (3.0)	6 (3.6)	2 (1.6)
TGFB1	rs1800469	GG		338 (58.3)	198 (58.8)	131 (59.8)	98 (60.1)	92 (56.8)	73 (62.9)
	(n=917)[a]	GA		198 (34.1)	120 (35.6)	77 (35.2)	57 (35.0)	56 (34.6)	39 (33.6)
		AA		44 (7.6)	19 (5.6)	11 (5.0)	8 (4.9)	14 (8.6)	4 (3.4)
	rs1800470	TT	Leu10Leu	297 (49.8)	180 (51.7)	118 (52.2)	86 (50.9)	82 (49.1)	67 (54.5)
	(n=944)[a]	TC	Leu10Pro	243 (40.8)	143 (41.1)	93 (41.2)	71 (42.0)	67 (40.1)	52 (42.3)
		CC	Pro10Pro	56 (9.4)	25 (7.2)	15 (6.6)	12 (7.1)	18 (10.8)	4 (3.3)
	rs2241718	GG		436 (73.8)	255 (74.1)	173 (77.6)	129 (77.7)	121 (72.9)	99 (81.1)
	(n=935)[a]	GA		138 (23.4)	82 (23.8)	46 (20.6)	36 (21.7)	42 (25.3)	21 (17.2)
		AA		17 (2.9)	7 (2.0)	4 (1.8)	1 (0.6)	3 (1.8)	2 (1.6)

[a]Number of successful genotyping analysis
